# The Evolution of Uroflowmetry and Bladder Diary and the Emerging Trend of Using Home Devices From Hospital to Home

**DOI:** 10.2196/66694

**Published:** 2025-01-28

**Authors:** Ming-wei Li, Yao-Chou Tsai, Stephen Shei-Dei Yang, Yuan-Hung Pong, Yu-Ting Tsai, Vincent Fang-Sheng Tsai

**Affiliations:** 1 Division of Urology, Department of Surgery Taipei Tzu Chi Hospital Buddhist Tzu Chi Medical Foundation New Taipei City Taiwan; 2 School of Medicine Tzu Chi University Hualien Taiwan; 3 Department of Urology Ten Chen Hospital Taoyuan City Taiwan; 4 PhD Program of Mechanical and Aeronautical Engineering Feng Chia University Taichung Taiwan; 5 Department of Urology National Taiwan University Hospital Taipei city Taiwan

**Keywords:** lower urinary tract symptoms, uroflowmetry, bladder diary, home devices, bladder, noninvasive, evaluations, viewpoint, diagnostic, mobile health

## Abstract

Although uroflowmetry and bladder diaries are widely used for noninvasive evaluation of lower urinary tract symptoms, they still have limitations in diagnostic capability and users’ convenience. The aim of this paper is to discuss potential solutions by reviewing (1) the evolution and current clinical use of uroflowmetry and bladder diary, including clinical guidelines, daily practice applications, and their historical development; (2) a growing trend toward using home devices with various technologies; and (3) a comprehensive comparison of the strengths and weaknesses of these home devices. In our opinion, the following points can be highlighted: (1) the emerging trend of using home devices can enhance diagnostic capabilities through repeated measurements and the convenience of at-home testing and (2) home devices, which provide both frequency-volume and uroflowmetry information, have the potential to transform the management of lower urinary tract symptoms.

## Introduction

As the population ages, there has been an increase in patients reporting lower urinary tract symptoms (LUTS) in recent years. To objectively assess the function of the lower urinary tract, uroflowmetry and bladder diaries (BD) are commonly used noninvasive examinations for those experiencing LUTS.

Uroflowmetry can measure various parameters during the voiding phase, including maximum flow rate (Qmax), and voided volume, which are essential metrics. Additional parameters, such as flow pattern, time to Qmax, flow time, and average flow rate, provide further insights [1]. This makes uroflowmetry a comprehensive and objective tool for evaluating the voiding phase. Consequently, uroflowmetry is recommended in the guidelines of both the American Urological Association and the European Association of Urology for assessing LUTS [2-4].

BD offers physicians valuable insights into urinary frequency, functional bladder capacity, most frequently observed bladder volume, urgency and urge urinary incontinence episodes, volume of water intake, nocturnal or daily urine output ratio, and associated bladder pain episodes [5]. They assess not only the storage phase but also some symptoms related to the voiding phase. For optimal adherence and reliability, a duration of 3-7 days is recommended for maintaining a BD [3,6]. Additionally, BD is included in the guidelines of both the American Urological Association and the European Association of Urology for evaluating overactive bladder and LUTS [2-4].

Even though both of them can provide physicians with several parameters for evaluating the lower urinary tract, there are still some limitations of uroflowmetry and BD for depicting the whole picture of patients’ LUTS, such as inconveniences, inadequate measurement frequency for uroflowmetry, lack of objective recording, and poor adherence for BD.

The aim of this paper is to discuss potential solutions for the earlier-mentioned limitations by reviewing (1) the evolution and current clinical use of uroflowmetry and BD, including clinical guidelines, daily practice applications, and their historical development; (2) a growing trend toward using home devices with various technologies; and (3) a comprehensive comparison of the strengths and weaknesses of these home devices.

## Clinical Application and Limitation of Uroflowmetry and BD

### Clinical Application and Limitation of Uroflowmetry

Uroflowmetry is typically performed in health care institutes, where patients may experience heightened emotional effects compared to when they are at home. Pre- and intratest anxiety (just like “white-coat hypertension”) should be taken into consideration for its effect on lowering test reproducibility [7]. This practice also causes inadequate measurement frequency and a lack of voiding in different scenarios.

As to representativeness, some parameters are prone to within-subject variation [8-11], so it is recommended to repeat uroflowmetry measurements. The diagnostic accuracy of uroflowmetry is largely affected by threshold values [12,13], especially with physiological compensatory processes, detrusor underactivity, or an underfilled bladder [14]. Although uroflowmetry can be used to monitor treatment outcomes [15] and to correlate symptoms with these objective findings [12,16], its clinical value is still limited, as it is unable to differentiate between the possible underlying mechanisms. Again, specificity can be improved by repeated flow rate testing [6]. However, it is not feasible for a patient to receive multiple uroflowmetry in a clinical setting. Therefore, Caffarel et al [17] and Bray et al [18] even suggested the use of home uroflowmetry to achieve multiple measurements to improve the reliability of uroflowmetry.

In summary, uroflowmetry should be performed in a comfortable environment repeatedly to represent the whole picture of a patient’s voiding pattern. This means that an at-home uroflowmetry is preferred.

### Clinical Application and Limitation of BD

BD provides a method of quantifying symptoms, such as frequency of urge incontinence events and number of nocturia episodes [3], and reduces recall bias. However, it is not completely objective because the process relies on manual actions, such as recording the time and using measurement tools. These will affect BD’s reliability.

Furthermore, the use of BD might induce a “bladder training” effect and influence the frequency of nocturnal voids [19]. Though it is important to guide patients to live an appropriate lifestyle [20], such modifications could potentially obscure the true micturition behavior of the patient. It is just like a clinical “uncertainty principle” (when you measure a system and change it at the same time), which should be taken into consideration when the goal of the test is to accurately depict the true picture of a patient’s micturition. It is preferred to record a BD intuitively and insensibly.

The duration of BD has to be long enough to reduce sampling errors but short enough to avoid nonadherence [21]. It is recommended to conduct a BD including at least 3 days (continuous or separated) [22]. However, a longer BD is generally more reliable [23-26]. Because of the considerable effort required to complete each entry in a BD, there has been minimal achievement in comparing and validating BDs. Until now, the International Consultation on Incontinence Questionnaire BD is the only one that has undergone full validation [27]. The current protocol of BD necessitates that patients physically collect and measure each void, recording the volume along with associated symptoms. This practice requires patients’ thorough intellectual understanding of the whole procedure and objectively recording BD. Among a survey of urogynecology or female pelvic medicine and reconstructive surgery specialists’ fellowship-trained attendings, 25% had never or rarely (frequency of use <20%) used BD, and 97.5% reported difficulties associated with obtaining correctly completed and clinically applicable BD. The authors mentioned the best way is to teach patients with thorough instruction [20]. Therefore, a BD system capable of automation, objective recording, and repeated measurement appears to be the ideal solution.

## Innovative and Possible Solutions for Avoiding Limitations

### Evolution History of Uroflowmetry

As discussed in the “Clinical Application and Limitation of Uroflowmetry” section, it is important to provide patients with a comfortable environment and repeated measurements. In a clinical setting, urine weight–based, dipstick, and spinning-disk uroflowmetry are currently the most common conventional measurements of flow rate. Urine weight–based uroflowmetry detects the rate of changed weight or volume of voided urine. Dipstick uroflowmetry uses a dipstick immersed in voided urine sensing the change of fluid height and obtaining the flow rate. Spinning-disk uroflowmetry relates the power needed to counteract the slowing speed of the spinning disk hit by falling urine to the rate of urine flow [28]. In this paper, some trials of home uroflowmetry (relatively comfortable environment and feasible for repeated measurements) will be presented and discussed.

There have been several types of funnel-based uroflowmetry in the market or research fields [29-36]. The principle of funnel-based uroflowmetry is a combination of a funnel and some calibrating assembly for measuring Qmax. They all need to be operated manually, and the results are then recorded on paper. One funnel-based home uroflowmetry has been developed for decades [37]. P-Flow (Tejnaksh Healthcare Ltd) is now a commercially available home uroflowmetry, which provides not only Qmax, average flow, voiding time of urine, flow curve, and total voided volume of urine but also simple urinalysis by an accompanied dipstick. However, users need to upload photos of the indicators on the device after finishing the test onto the health care company’s server for interpreting the final results. There would be a time lag before obtaining the interpretation from the experts of this company. Additionally, it was found that Tejnaksh, the company behind this product, is a recognized teaching institute by the Ministry of Health and Family Welfare in India. To our knowledge, however, the product itself has not obtained certification from any other health authority.

There are at least 2 series of active research groups devoted to sound-based uroflowmetry, one from Singapore [38] and another from Korea [39]. Both systems use mobile phones to detect voiding sound and transfer it to the signal of voiding. Important urodynamic parameters, such as Qmax, voided volume, voiding time and average flow rate, and voiding flow patterns, can be presented through the embedded artificial intelligence (AI) algorithm. ProudP is currently commercially operated in the United States, which not only enhances the communication between patients and doctors but also improves the care quality for patients with LUTS. However, sound sensing uroflowmetry may be interfered in some noisy environments and even by the material of the testing urinal. There will be more discussion about the features of home devices in the “Comparison Between Home Devices of Different Technologies” section.

To provide a comfortable and intuitive environment for uroflowmetry, a toilet-shaped uroflowmetry (UM-100, Toto Ltd) was commercially developed in 2008 and was initially designed for clinical use [40]. A water-level measurement unit is installed with a connection to the toilet bowl. By balancing the hydraulic pressure between the measurement unit and the toilet bowl (communicating tubes in physics), urination-caused change in the water level can be measured and transformed to uroflowmetry parameters. The merit of this system is no necessity to do any cleaning work after each measurement. However, since the system detects the change in the total volume, it may have erroneous measurements when patients pass stool and urinate simultaneously [41].

A novel technology of vibration-based uroflowmetry was developed by a Taiwan-based interdisciplinary team [42]. This system simultaneously detects vibration signals during urination using an accelerometer alongside conventional uroflowmetry. Strong correlations were observed between this system and conventional uroflowmetry for parameters such as Qmax, voided volume, voiding time, and time to Qmax. Additionally, an AI model was used to analyze and predict 6 predefined patterns of uroflow curves, aiding in diagnosing voiding dysfunction with an accuracy of approximately 98%. This relatively low-cost system is suitable for automatic home urinary monitoring and enables repeated uroflow monitoring of patients outside health care institutions.

### Evolution History of BD

With the reflection in the “Clinical Application and Limitation of BD” section, it is suggested to provide patients with automation, objective recording, and repeated measurements. It can be traced back to 1993 when the first computerized voiding diary “Compu-Void” was developed [43]. Compu-Void was a 64,000 RAM–capacity manual unit operated on the primitive operating system “DOS” of a personal computer. In the following decades, numerous trials have been conducted in pursuit of enhancing BD, aiming for automation, objective recording, and repeated measurement.

Quinn et al [44] ever developed a logical flow for asking users’ symptoms on the portable electronic BD “MiniDoc.” The advantage of an electronic data-inputting device lies in its accuracy and speed in retrieving data for further analysis [44]. Mangera et al [45] compared a paper-card reader and a hand-held input device with conventional written BD and found that an intuitive and user-friendly interface led to not only patients’ preference but also the efficiency and accuracy of data management. With the onset of the digital era, application software (apps) on mobile phones and tablet computers began to play a crucial role in recording BD [46], and their numbers surged rapidly. Reports indicated that there were 55 apps available in languages such as Portuguese, Spanish, French, or English, with some offering functions for analyzing incontinence episodes and nocturia [47]. However, all of these apps require manual input.

For automated entry of data, Takai et al [48] ever introduced a body weight–based BD, which demonstrated a strong correlation between the voided urine weight recorded by the device and voided urine weight measured manually by the examinee [48]. This is the best practice of frequency or volume record and should be combined with entering episodes of LUTS to accomplish a comprehensive BD, though it cannot measure accurate urine weight during defecation.

With robust procedures of BD, the most common way to teach patients how to complete a BD is by providing detailed instructions along with any type of BD. In the current digital age, it should be expected that more clinicians like to use mobile apps or other digital resources. However, it was shown that very few clinicians actually used mobile apps (0.9%) or directed patients to use internet resources (1.2%) [20]. Furthermore, there is still no evidence to suggest that these digital apps can independently improve return rates or accuracy of the BD. In our opinion, the reason behind this is that there is only little difference between paper-based and electronic-based BDs. Electronic BDs simply record data through manual input or touch-screen processes, lacking automation. Moreover, the most cumbersome step of BD, collecting and measuring urine volume, remains unchanged. In this regard, a body weight–based BD offers certain advantages [48].

Since some important characteristics of a reliable BD, such as automation, objective recording, and repeated measurement, are fulfilled by the earlier-mentioned technologies (Table 1), we can envision a more advanced tool for accurately depicting a patient’s voiding patterns. Additional suggestions for the development of a combined home uroflowmetry and BD system are as follows: elimination of the need for cleaning after each use, incorporation of an intuitive and user-friendly interface, integration of wireless communication capability, accessibility of data for both users and doctors, and suitability for both storage and voiding phases. Therefore, such a comprehensive and intuitive “voiding recorder” will be a new standard for at-home voiding monitoring.

**Table 1:** Chronology of uroflowmetry and bladder diary (BD) advanced technologies in the recent century.

Year	Event	Remarks
1932	Ballenger suggested the maximum distance of a man’s urine ejecting	[49]
1948	Drake recorded change in urine weight with time and manually calculated flow rate	[49,50]
1957	Kaufman improved Drake’s system with electrical apparatus	[51]
1957	Von Garrelts electrically calculated flow rate	[41]
1965	Smith designed funnel-based uroflowmetry	[29]
1976	Drach used a dipstick to estimate the flow rate	[35]
1993	The first computerized voiding diary “Compu-Void” was developed	[43]
1999	International Continence Society defined uroflowmetry parameters	[52]
2003	Quinn drew a logical flow for asking users’ symptoms	[44]
2008	Toto developed communicating tube uroflowmetry	[40]
2014	Mangera used paper-card reader	[45]
2014	Bright designed the first standardized and validated BD (ICIQ^a^ BD)	[27]
2015	Krhut compared uroflowmetry and sonouroflowmetry	[53]
2016	Application of mobile phones and tablet computers in recording BD	[46]
2021	Takai introduced a body weight–based BD	[48]
2022	Pong linked vibration with uroflowmetry	[42]

^a^ICIQ: International Consultation on Incontinence Questionnaire.

## The Emerging Trend of Using Home Devices

As we enter the era of AI, big data become crucial for training and applying AI models. This enhances the value of home devices, particularly for their ability to repeatedly measure home uroflowmetry and generate big data. Home devices facilitate mobile health or medicine by offering a comfortable environment, enabling repeated measurements, and providing big data for communication and application. It has been noted that extensive at-home data are often more reliable than single in-office tests [54]. Recently, there has been a surge in interest in home uroflowmetry at international scientific meetings and in literature [9,55-58]. Several commercial home uroflowmetry devices have also been introduced, offering repeated measurements of voiding parameters and uroflow curves. This method of repeated home measurement can provide a comprehensive picture of patients’ daily-life voiding without the anxiety and stress associated with office uroflowmetry tests [9,55]. As mentioned in the “Introduction” section, uroflowmetry primarily describes the condition of the voiding phase, while BD addresses the storage phase and some symptoms related to voiding. Each can be applied to patients with different types of voiding dysfunction. Following advancements, home devices can now measure parameters in both the storage and voiding phases, making them suitable for almost all patients with voiding dysfunction.

Certainly, there is a need for home uroflowmetry to offer repeated measurements and convenience, addressing several clinical and practical challenges for practicing urologists [54]. With various home uroflowmetry technologies emerging in the market, it is important to understand their strengths and weaknesses. What are the comparative advantages and disadvantages of these different home uroflowmetry technologies?

## Comparison Between Home Devices of Different Technologies

All types of home uroflowmetry technologies share common strengths, such as automatic recording, repeated measurements, and the generation of big data. However, each technology also has its own unique strengths and weaknesses when used for home uroflowmetry. Table 2 outlines various features of these technologies, including accuracy, susceptibility to environmental interferences, uroflow curve pattern recognition, AI algorithms, and contact-free operation.

**Table 2:** The comparison on different technologies for home uroflowmetry.

Features or technologies	Weighing (gravimetric)	Height sensor	Sound	Vibration
Accuracy	FDA^a^ certified [57]	FDA certified [59]	Correlation with office uroflowmetry (*R*=0.91) [54]; prediction rate of 99% [60]	Uroflow curve pattern recognition accuracy>0.98 [42]
Vulnerability to the surrounding interferences	No	No	Yes [61]	No
Uroflow curve pattern recognition	No	No	Yes [60]	Yes
AI^b^ algorithm or model	No	No	Yes [60,61]	Yes
Contact-free (no need for installation or cleaning)	No	No	Yes	Yes

^a^FDA: Food and Drug Administration.

^b^AI: artificial intelligence.

In terms of accuracy, all technologies yield results comparable to office uroflowmetry, with some even receiving Food and Drug Administration approval. However, sound-based technologies are sensitive to environmental interferences like noise or barriers due to their measuring mechanism. For accurate recording, the urine stream must be voided into a water-filled commode rather than a urinal [54]. Additionally, the sound of voiding varies between men and women, likely due to anatomical and postural differences during urination. Men, who typically urinate standing up, produce louder sounds, as there are no barriers to dampen the sound. In contrast, women, who usually urinate sitting down, produce quieter sounds, and the sitting position can block sound transmission. To address these issues, different models have been developed for both genders [61].

Vibration-based technology offers a potential solution by reducing the barriers to sound transmission. Vibration is transmitted more directly than sound, which travels through the air, while vibration is conveyed through the concrete toilet bowl or urinal to the sensor. Consequently, sound-based technologies are more vulnerable to surrounding noise compared to vibration-based ones [56].

After reviewing the literature, it is evident that only sound- and vibration-based technologies have used AI models or algorithms for uroflow curve pattern recognition [42,60,61]. These 2 technologies are advantageous for intuitive and contact-free measurements, as they do not require installation or cleaning for each use. The calibration of any device is crucial and must be verified during the approval process. It should be ensured that all home devices receive approval from health authorities before clinical application. Additionally, with repeated measurements using home devices, the current reference ranges for clinical urodynamic studies may be revised accordingly. However, such revisions should be undertaken only after sufficient evidence has been accumulated. These home devices not only enhance accessibility for patients and physicians in assessing voiding patterns, but they also improve the diagnosis and treatment process by collecting more comprehensive information. The use of home devices is anticipated to transform the management of LUTS.

## Conclusions

Uroflowmetry and BD are key diagnostic tools for LUTS, despite facing limitations such as the absence of a comfortable environment and infrequent measurements for uroflowmetry as well as the lack of automation, objective recording, and repeated measurement for BD. Technological advancements have addressed some of these limitations.

In our opinion, the following points can be highlighted: (1) the emerging trend of using home devices can enhance diagnostic capabilities through repeated measurements and the convenience of at-home testing and (2) home devices, which provide both frequency-volume and uroflowmetry information, have the potential to transform the management of LUTS.

## Abbreviations

AI: artificial intelligence
BD: bladder diary
LUTS: lower urinary tract symptom
Qmax: maximum flow rate
